# Development and psychometric evaluation of a pneumatic tourniquet work standards scale

**DOI:** 10.1186/s13018-024-04920-8

**Published:** 2024-07-26

**Authors:** Hamideh Fanoudi, Camellia Torabizadeh, Mahnaz Rakhshan, Gholam Hossain Shahcheraghi

**Affiliations:** 1 School of Nursing and Midwifery, Shiraz University of Medical Sciences, Shiraz, Iran; 2 Community Based Psychiatric Care Research Center, School of Nursing and Midwifery, Shiraz University of Medical Sciences, Shiraz, Iran; 3 Department of Orthopaedics, School of Medicine, Bone and Joint Disease Research Center, Shiraz University of Medical Sciences, Shiraz, Iran

**Keywords:** Operating room, Orthopedic procedure, Pneumatic tourniquet, Psychometrics

## Abstract

**Objective:**

Pneumatic tourniquets are among the most essential equipment for controlling bleeding in orthopedic surgeries. However, incorrect application of pneumatic tourniquets is accompanied by many hazards and complications for patients. Evaluation of surgical teams’ use of pneumatic tourniquets and establishment of protocols can play an important role in improving patient safety, minimizing risks, and increasing the lifetime of this equipment. Accordingly, the present study was conducted to develop and assess the psychometric properties of a pneumatic tourniquet work standards scale.

**Methods:**

The present study is a methodological work carried out in two stages. In the first stage, an initial version of the scale was developed based on existing research and panel reviews. In the second stage, the psychometric properties of the scale were tested in terms of face validity (measured qualitatively and quantitatively), content validity (measured qualitatively and quantitatively), item analysis, construct validity, and reliability (internal consistency and stability).

**Results:**

The initial version of the scale consisted of 91 items. After several meetings of the research team, the number of items decreased to 81. In the course of face and content validity testing, 40 items were eliminated, leaving 41 items on the scale when it entered the construct validity testing stage. For evaluation of construct validity, a sample of 300 operating room nurses was recruited. The Exploratory Factor Analysis (EFA) results showed a structure supported by seven factors and 41 items. The reliability of the scale was confirmed by internal consistency analysis, with a good Cronbach’s alpha (0.85), and test–retest analysis, with good values of ICC (0.95).

**Conclusion:**

The present instrument is a reliable and valid scale which fills the gap in assessment of surgical team members’ use of pneumatic tourniquets. The developed scale can be employed by researchers and managers of medical centers to identify hazards in applying pneumatic tourniquets and devise educational programs to eliminate or reduce the existing issues.

## Introduction

Maintaining patient safety by preventing any injuries to the patient during clinical interventions is one of the indexes of the quality of healthcare and a major concern in the healthcare system [1, 2]. In developed countries, one in every ten patients who receive hospital care is affected by medical errors, and in developing countries, that rate is much higher [1]. Among hospital units, the operating room is a complex environment where the probability of medical errors is high [2]. Increasing use of technology in surgeries has increased the complexity of surgical procedures, raising the incidence of medical errors [3]. One of the major concerns in surgeries is bleeding, and there are a variety of methods for controlling it, including application of pneumatic tourniquets [4, 5].

Every day, pneumatic tourniquets are used in over 15,000 surgeries all over the world [6]. The U.S. Food and Drug Administration has rated pneumatic tourniquets as class I medical devices [7]. In knee arthroplasty, longer application of tourniquets is associated with shorter length of surgery, reduction in intra-operative blood loss, higher post-operative haemoglobin levels and less need for blood transfusion [8]. These devices have many advantages, including giving surgeons a better field of view and decreasing the rate of bleeding and the length of surgeries; however, certain precautions should be taken in using this equipment [7, 9].

Incorrect application of pneumatic tourniquets can lead to neuromuscular injuries (40%) [10], limb paralysis with full recovery (68%) and partial recovery (18%), post-tourniquet pain (50%), chemical burns (16%), and post-tourniquet syndrome (15%) [11]. Among other potential consequences of using pneumatic tourniquets are the compartment syndrome, pressure sores, pulmonary embolism, rhabdomyolysis [7], DVTs, wound infection, and delayed healing [12]. According to a systematic review, nerve injuries caused by tourniquets can happen over a wide spectrum of times and pressures [13]. These complications can occur as a result of low pressure, inadequate exsanguination, selection of the wrong cuff, looseness of the cuff, slow inflation of the cuff, or deflation of the cuff in the course of surgery [14, 15]. Using a tourniquet throughout surgery can reduce hemorrhage to a great degree, but there are many safety measures which must be complied with [12]. A study in Turkey found that there are many variations in using tourniquets among orthopedic surgeons. These differences, especially in the case of unsuitable pressure of tourniquets and inadequate training in using these devices, can lead to major complications. Appropriate education is the key to providing quality care to patients. In addition, standard protocols on using pneumatic tourniquets must be developed [16].

The Association of perioperative Registered Nurses (AORN) recommends that the members of surgical teams must be educated so that they possess enough knowledge and skill in using pneumatic tourniquets [17]. The latest practical guidelines on using pneumatic tourniquets issued by AORN address indications and contraindications of pneumatic tourniquets, preoperative evaluation, application and inflation of the cuff, monitoring, and postoperative pain and swelling control following the application of pneumatic tourniquets [14, 18]. A review of these guidelines shows that they do not cover all the topical aspects of these devices; moreover, the sheer number of the guidelines increases the probability of healthcare professionals’ forgetting many of them while using pneumatic tourniquets. On the other hand, the existing executive guidelines are simply a set of standard recommendations and there is no guarantee that they are followed by surgical teams in practice.

A review of literature showed that, with regard to application of tourniquets, instruments developed by Albaker et al. (2023) [10], Ajibade et al. (2021) [11], Boya et al. (2016) [16], Yalcinkaya et al. (2014) [19], and Cunningham et al. (2013) [20] were available.

Albaker et al. (2023) developed a 17-item scale in Saudi Arabia using the AORN guidelines and questions from the study by Yalcinkaya et al. to assess orthopedic surgeons’ knowledge. The items on this scale address the location of the cuff, limb occlusion pressure, two cases where tourniquets are not to be used, prevention of the prep solution leaking to under the cuff, type of padding, and calibration of the device [10]. Ajibade et al. (2021) developed a self-administered questionnaire to assess the knowledge of orthopedic surgeons and residents in Nigeria. Eight items on the questionnaire concern the placing and the person in charge of applying the tourniquet, padding, maximum length of using the tourniquet, and the complications of using tourniquets. This study also found that, in addition to surgeons and residents, tourniquets are applied by other members of the surgical team, including plaster technicians and perioperative nurses. This finding underlines the significance of developing a general instrument for all the members of the surgical team [11].

The 18-item questionnaire developed by Boya et al. (2016) in a pilot study in Turkey measures the knowledge of orthopedic surgeons, residents, and faculty members. Ten items on the questionnaire are about the padding under the tourniquet, location of the cuff, pressure of the tourniquet, time of administering antibiotic prophylaxis before applying the tourniquet, maximum length of using the tourniquet, cases where tourniquets are not to be used, and the principles of recording observations. The other 8 questions addressed descriptive data, including deciding what type of tourniquet to use, type of cuff, the care provider in charge of applying the tourniquet, experience of observing tourniquet use complications, and receiving periodic educational practice. In view of the many variations in opinions about applying pneumatic tourniquets, Boya suggests an extensive literature review toward developing standard protocols [16]. In the 12-item questionnaire developed by Yalcinkaya et al. (2014) in Turkey, 8 items measure orthopedic surgeons’ knowledge of pneumatic tourniquets and 4 items address the respondents’ demographics. The questions concern issues and complications in applying tourniquets, type of padding, limb exsanguination, experience of observing post-tourniquet use complications, preferred cuff pressure (CP), and tourniquet inflation time (TIT) [19]. Cunningham et al. (2013) developed a 15-item scale in Ireland to measure orthopedic surgeons’ knowledge of pneumatic tourniquets. Seven items on this scale concern limb exsanguination, type of cuff, extent of pressure, maximum duration of using a tourniquet and four items address issues and complications which may follow application of tourniquets. As a limitation of their study, the researchers mentioned that they did not take many aspects of using tourniquets into account, e.g. the characteristics of a suitable tourniquet and its accessories, the padding under the cuff, and the duration of using a tourniquet for different individuals. Their study also found that, though usually orthopedic surgeons fasten the tourniquet cuffs themselves, in some cases the cuffs are applied by anesthesiologists or nurses [20].

The existing questionnaires fail to address certain important aspects of safe application of pneumatic tourniquets in practice. Thus, the available instruments are limited in scope and do not cover all the dimensions of pneumatic tourniquet application protocols. Moreover, these instruments generally assess the surgeons’ knowledge, while studies report that it is common for tourniquets to be applied by other members of the surgical team. According, there is a need for a general instrument which can be used by all the members of the surgical team. Another shortcoming of the existing instruments is that they did not go through rigorous psychometric validation. Identification and prevention of hazards in using pneumatic tourniquets, which can minimize the rate of errors and enhance patient safety, require an instrument which is valid, reliable, and transferrable. Thus to fill the gap in the available theoretical and practical knowledge, the researchers conducted a study to develop and assess the psychometric properties of a pneumatic tourniquet work safety standards scale.

The present study is the first attempt at developing a comprehensive pneumatic tourniquet work standards scale which went through rigorous psychometric validation designed for all the members of the surgical team. Employment of this scale can help prevent complications associated with incorrect use of pneumatic tourniquets, thereby contributing to patient safety and the practice of the surgical team.

## Background

In hospitals in Iran, operating room nurses are in charge of using pneumatic tourniquets. Though tourniquet cuffs are placed by surgeons, residents, or nurses, operating room nurses are responsible for other precautions in using tourniquets before, during, and after surgery. An instrument specifically designed to evaluate the surgical teams’ performance in applying pneumatic tourniquets has not been developed yet. Workshops on using pneumatic tourniquets, which are part of medical equipment training programs, are only for operating room personnel and are held once a year. However, attending these workshops is not compulsory for surgeons and operating room personnel. Moreover, these workshops do not address all the details about application of pneumatic tourniquets and only provide a brief description of the settings of the devices: the workshops do not deal with such considerations as testing the working condition of the device, safety measures before, during, and after surgery, the principles of recording observations, and the possible complications of pneumatic tourniquets. In their 4-year undergraduate programs, operating room nurses receive only two 2-hour sessions of theoretical education in application of pneumatic tourniquets. In the present study, the research team witnessed many instances of incorrect application of pneumatic tourniquets and the resulting consequences. Accordingly, the present study was conducted to develop and measure the psychometric properties of a pneumatic tourniquet work standards scale.

## Research questions

1. What are the key components of a comprehensive pneumatic tourniquet work standards scale?
2. How valid is the developed scale in measuring the work standards of pneumatic tourniquet usage?
3. How reliable is the developed scale in measuring the work standards of pneumatic tourniquet usage?

## Materials and methods

The study was conducted in two stages: [1] scale development and [2] psychometric evaluation: refining the scale and evaluating its psychometric properties.

### Scale development

In the first stage of the study, toward item generation, an initial pool of items was developed based on 2 sources: [1] a comprehensive literature review and [2] expert consensus. To develop the items of the scale, the researchers conducted an extensive review of literature (articles, theses, and books) available at databases and the websites of related organizations, including the Association of Surgical Technologists and the AORN, and Medical Device Reporting. In the present study, all the articles published in English, regardless of the time of their publication, in the databases of PubMed, Science Direct, Google Scholar, Scopus, Medline, Elsevier, CINAHL, ProQuest, Thomson Reuters, Embase, and UpToDate were extracted by one of the authors. To ensure exhaustive coverage of the subject, the initial search addressed titles, keywords, and abstracts using different combinations of keywords, such as ‘pneumatic tourniquet’, ‘tourniquet’, ‘tourniquet application’, ‘safety’, ‘hazard’, ‘questionnaire’, ‘operating room’, ‘nurse’, ‘surgery’, ‘psychometric evaluation’, and ‘side effects’ and Boolean Operators (AND and OR). The keywords extracted from MeSH and related articles were used for appropriate syntax search in each database. Also, the grey literature on organizational websites, e.g. the AORN and WHO, was examined. Next, two of the authors examined the titles, abstracts, and keywords of the selected articles. Any disagreement over the selection of articles was discussed by all the authors until agreement was reached. The inclusion criteria for the articles were having been released in English and being relevant to the objective of the study. The articles whose full texts were not available were excluded.

Subsequently, in several meetings with a panel of experts, the researchers eliminated some of the items and the final list of the items was prepared for the next stage. The content analysis of literature and panel reviews for item generation assisted in the formulation of the systematized concept and thus provided with the content evidence of validity for the instrument.

### Psychometric evaluation

In the second stage of the study, the psychometric properties of the developed scale were measured. Generally, for psychometric evaluation of instruments, the two indexes of reliability and validity are measured. According to Hugan, to determine the validity of an instrument, its face validity, content validity, construct validity, criterion validity, and predictive validity must be calculated [21]. As for reliability, DeVellis and Thorpe recommend measuring test-retest reliability, parallel forms reliability, and internal consistency [22]. In the present study, the validity of the instrument was measured using methods of qualitative and quantitative evaluation of face validity, content validity, and construct validity. The reliability of the instrument was measured by calculating its internal consistency and stability.

### Participants

In different stages of evaluation of the validity of the scale, the researchers used panels of experts. The members of the review panel were operating room nurses, operating room department faculty members, instrument development experts, orthopedic surgeons, and medical equipment technicians. The inclusion criteria for these individuals were as follows: willingness to participate, familiarity with the concept under study, knowledge and experience of using pneumatic tourniquets, and expertise in developing instruments.

For assessment of construct validity, the researchers used a population of operating room nurses because, in Iran, operating room nurses are responsible for placing and monitoring pneumatic tourniquets. The inclusion criteria were being a circulating or scrub nurse, having at least six months’ experience of practice in the operating room, and being willing to participate in the study. The nurses whose workplaces changed or did not answer all the items on the scale were excluded.

### Validity analysis

#### Face validity

For qualitative measurement of face validity of the scale, four operating room nurses, four operating room department faculty members, two instrument development experts, four orthopedic surgeons, and one medical equipment expert were interviewed on a face-to-face basis. Based on the panel of experts’ comments, the items were revised or eliminated. For quantitative measurement of face validity, 15 operating room nurses were selected and asked to rate the significance of each item on a 5-point Likert scale. The impact score (IS) of each item was calculated accordingly. The items with an impact score of greater than 1.5 were considered acceptable and were retained for the next stage [23].

#### Content validity

For qualitative measurement of content validity of the scale,15 experts, including two instrument development experts, and 13 faculty members were asked to rate the items in terms of syntax, wording, placement of the items, and scoring. As for quantitative measurement of content validity, the researchers calculated the content validity ratio (CVR), content validity index (CVI), and scale content validity index (S-CVI) of the scale.

For calculation of CVR, 15 experts familiar with application of pneumatic tourniquets rated the necessity of the items. Following the Lawshe table, the items with a CVR of greater than 0.49 were considered to be significant (*P* < 0.05) and were retained [24]. In order to measure CVI, the researchers used Waltz and Basel’s approach and had 15 experts rate the items in terms of relevance, clarity, and simplicity [25]. The items with a CVI of greater than 0.79 were retained, the items with a CVI of between 0.70 and 0.79 were considered questionable and had to be revised, and the items whose CVI was smaller than 0.70 were eliminated [26]. The S-CVI of the instrument was measured using two indexes: Scale Content Validity Index/Universal Agreement (S-CVI/UA) and S-CVI/Ave. To measure S-CVI/UA, the researchers calculated the ratio of the items which had been assigned a score of 3 or 4 by all the experts to the sum of the items. By agreement, the value of S-CVI was found to be 0.7 [27]. S-CVI/Ave was calculated by adding the CVI of all the items and dividing the sum by the number of the items. The level of acceptance of S-CVI/Ave is 0.8 [28].

In this stage, the kappa coefficient was measured too. The kappa coefficient is a measure of inter-rater agreement over the relevance or non-relevance of items [26]. In the present study, the kappa statistic was calculated without taking chance agreement into account. A kappa of 40–59% is regarded as relatively good, and a kappa of 60–74% is considered to be perfect [29].

#### Construct validity

The construct validity of an instrument can be measured in terms of a variety of indexes, including convergent validity, discriminant validity, EFA, and confirmatory factor analysis (CFA) [30, 31]. In the present study, to verify the construct validity of the scale, the researchers conducted EFA. EFA with principal component extraction was used to determine the factors, reduce the items, and examine the factor structure. For assessment of construct validity, the researchers used a sample of 300 operating nurses who were in practice in teaching hospitals. The nurses were selected by convenience sampling. In EFA, at least 4 to 10 subjects per item are required [26]. The present scale consisted of 41 items and 7 subjects were selected per item.

### Reliability analysis

To determine the reliability of the scale, the researchers applied the methods for measuring internal consistency and stability.

#### Internal consistency

The internal consistency of the scale was measured by calculating Cronbach’s alpha and the correlation between the even- and odd-numbered items. A Cronbach’s alpha of between 0.7 and 0.8 indicates satisfactory internal consistency [30]. To measure the internal consistency of the scale, the researchers used a sample of 300 operating room nurses to determine the Cronbach’s alpha of each factor and the whole scale.

#### Test-retest reliability

The stability of the instrument was measured via the test-retest method. Accordingly, the scale was completed twice by 30 operating room nurses with a set interval. Gray et al. recommend a two- to four-week interval between the two tests [31]. In the present study, the interval was set at two weeks. Next, the correlation between the two stages was measured using the intraclass correlation coefficient (ICC) index. A correlation of greater than 0.75 is considered as satisfactory [32].

### Ethical considerations

The present study was carried out in compliance with ethical considerations: informing the participants about the objectives of the study before their participation, keeping the participants’ information confidential, assigning codes to the questionnaires, and excluding the subjects who are not willing to participate. The study was approved by an ethics committee.

## Results

The initial draft of the scale comprised 91 items. After evaluation by the research team, 10 items were found to overlap with the other items and were thus eliminated. The remaining 81 items were evaluated in the next stage. In evaluation of face validity, 15 items were merged and 15 other items were deleted because they overlapped with other items. Thus, the number of items decreased to 51. In quantitative evaluation of face validity, all the 51 items earned an impact score of above 1.5 and were thus retained.

In qualitative evaluation of content validity, three overlapping items were merged into one item, reducing the number of items to 49. Measurement of CVR showed that four items had a score of less than 0.49 and were thus eliminated. In calculation of CVI, all the remaining 45 items earned a score of above 0.79 and none of them was eliminated. The S-CVI/UA and S-CVI/Ave of the scale equaled 0.93 and 0.99 respectively. In the stage of item analysis, four items earned a total score of less than 0.3 and were thus eliminated. Next, the remaining 41 items were evaluated for construct validity.

To determine construct validity via EFA, a cross-sectional study was conducted on a sample of 300 operating room nurses from various hospital departments. The majority of the nurses were female (67.7%), had a bachelor’s degree (87%), and were permanent employees (63%) (Table 1).

**Table 1:** Demographics of the participants

Variable		Absolute frequency	Relative distribution (%)
Age (years)	23_30	134	44.7
	31_39	102	34.0
	40_49	47	15.7
	over 50	17	5.7
Gender	Male	97	32.3
	Female	203	67.7
Marital status	Married	196	65.3
	Single	104	34.7
Education	Associate degree in operating room nursing	21	7.0
	Bachelor’s degree in operating room nursing	261	87.0
	Master’s degree in operating room nursing	18	6.0
Type of employment	Permanent	189	63.0
	Contractual	47	15.7
	Trainee	64	21.3
Work experience (years)	0–3	87	29.0
	4–10	82	27.3
	11–15	58	19.3
	Over 16	73	24.3

The factor construct of the questionnaire was evaluated based on Kaiser-Meyer-Olkin (KMO) sampling index and Bartlett’s test of sphericity, main component analysis, scree plot and Varimax rotation. The sampling adequacy index of KMO was calculated and found to be 0.801, which was adequate and satisfactory. In addition, Bartlett’s test of sphericity was used to ascertain whether performing a factor analysis based on the matrix under study was justified and appropriate. The result of Bartlett’s test was significant (*P* < 0.000). The test showed the Chi-Square to have an approximate value of 3409.814 with a degree of freedom of 820 at *p* < 0.000 (Table 2).

**Table 2:** The results of the Kaiser-Meyer-Olkin (KMO) test and Bartlett’s test of sphericity

Bartlett’s test	Kaiser-Meyer-Olkin’s criterion for testing sampling adequacy	0.801
	Chi square	3409.814
	Degree of freedom	820
	Level of significance (P)	*P* < 0.000

In the next stage, EFA was conducted using principal component extraction and varimax rotation. In the present study, a factor loading of 0.4 was set as the minimum acceptable degree of correlation between each item and the extracted factors. At this point, the items which significantly correlated with each other were put in the same category. The results showed that the factor loadings of the items ranged between 0.334 and0.722, all of which were significant.

**Fig. 1:**
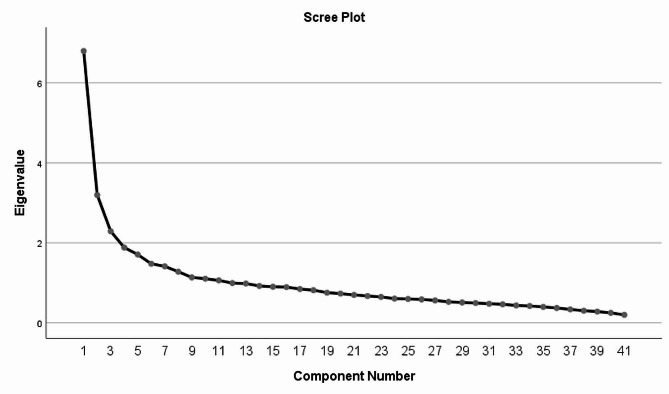
The factor analysis scree plot

To determine the number of factors in the questionnaire, the researchers used initial Eigenvalues and scree plot. Based on the scree plot, seven factors were identified for the questionnaire (Fig. 1): these factors accounted for 45.762 of the observed variance. In this stage, none of the items was eliminated and all the remaining 41 items were retained. The final version of the scale had 41 items, which were classified into seven factors: testing the functioning of pneumatic tourniquets (5 items), contraindications for the use of pneumatic tourniquets (4 items), considerations on selecting the right cuff (3 items), safe application of pneumatic tourniquets before surgery (11 items), during surgery (5 items), and after surgery (4 items), and record-keeping and documentation (9 items). Table 3 shows the factor loading of each dimension after the Varimax rotation.

**Table 3:** Factor loading of the seven factors

No.	Item	Extracted factors						
		1	2	3	4	5	6	7
**Subscale 1**: testing the functioning of pneumatic tourniquets								
1	I perform the periodic calibration of the central and portable tourniquets as recommended by the manufacturer.	0.430						
2	I check the cuff and connective tubing for leaks and make sure that pressure remains unchanged on the monitor.	0.446						
3	The connective tubing and power cable are not excessively stretched and are not in the way of the members of the surgical team.	0.453						
4	I set the monitor and audio alarms in such a way that the surgical team become aware of any changes.	0.555						
5	I disinfect reusable cuffs when they become contaminated with blood and other bodily discharges according to the instructions given by the manufacturer.	0.540						
**Subscale 2**: contraindications for the use of pneumatic tourniquets								
6	I examine the limb for nerve injuries (sensory and physical test of the limbs).		0.671					
7	I consider peripheral venous and blood circulation disorders (venous thromboembolism and compartment syndrome).		0.722					
8	I consider vein grafts, fistulas, and venous access ports in limbs.		0.654					
9	Before placing the tourniquet cuff, I check the patient’s skin for any injuries, e.g. blisters, bruises, and necrosis.		0.383					
**Subscale 3**: considerations on selecting the right cuff								
10	I consider the size and shape of the limb and the age of the patient to select the right cuff.			0.597				
11	I choose the length of the tourniquet cuff in such a way that at least 7.5 cm and at most 15 cm of the cuff overlaps.			0.638				
12	For overweight patients (BMI > 30), I use connector cuffs which are suitable for cone-shaped limbs.			0.558				
**Subscale 4**: safe application of pneumatic tourniquets before surgery								
13	I examine distal pulses to the tourniquet cuff.				0.369			
14	Before fastening the cuff around a limb, I inform the anesthesiologist.				0.550			
15	For padding, I use at least two layers of wrinkle-free padding which are wider than the tourniquet cuff.				0.609			
16	I fasten the cuff at the most muscular part of the proximal section of the limb.				0.564			
17	While disinfecting the limb, I am careful that the prep solution does not get under the cuff.				0.453			
18	I am careful that antibiotic prophylaxis is performed 5 to 20 min before the tourniquet cuff is inflated.				0.655			
19	After the administration of anesthetics and before the surgical incision is made, I inform the surgeon of the tourniquet pressure.				0.448			
20	I consider the cases when an Esmarch bandage cannot be used, e.g. due to infection, tumors, clots, and fractures.				0.541			
21	Before inflating the cuff, I perform exsanguination by raising the limb or using an Esmarch bandage.				0.512			
22	In the absence of an Esmarch bandage, I am careful to observe the 90-degree angle for upper limbs, 45-degree angle for lower limbs and 5-minute confidence time during exsanguination.				0.572			
Note: Among the three methods below this item, first select the method you most frequently use and then rate your choice form always to never.								
23	To set the tourniquet pressure: □ I always use the same pressure: Upper limbs: 200_250 mmHg Lower limbs: 250_350 mmHg □ I use a pressure higher than the patient’s systolic blood pressure (SBP): Upper limbs: SBP + 100 mmHg Lower limbs: SBP + 100–150 mmHg □ I use a pressure higher than limb occlusion pressure (LOP): LOP < 130 mmHg + 40 mmHg 131 < LOP > 190mmHg + 60 mmHg LOP > 191 mmHg + 80mmHg Pediatric Patients + 50 mmHg				0.470			
**Subscale 5**: safe application of pneumatic tourniquets during surgery								
24	I inform the surgeon and anesthesiologist about the maximum time the cuff can be inflated: Adults: 60 to 90 min. for upper limbs, 90 to 120 min for lower limbs Children: 60 to 75 min.					0.641		
25	In case of bleeding in the surgical site, I check the set pressure and the possibility of a leakage.					0.609		
26	I consider the patient’s physiological reactions (e.g. blood pressure and heart rate) to inflation of the cuff.					0.697		
27	If I should deflate the cuff during surgery, I consider the 10 to 15-minute time period.					0.334		
28	When I am deflating the cuff, to avoid sudden decrease in the patient’s blood pressure, I use the “sec stop” button (100 mmHg reduction in pressure every 5 s.) on portable tourniquets. On central tourniquets, I slowly decrease pressure to zero.					0.602		
**Subscale 6**: safe application of pneumatic tourniquets after surgery								
29	After deflating the tourniquet cuff, I check the patient’s vital signs.						0.718	
30	After deflating the cuff, I check distal pulses in the patient’s limb to make sure that blood is circulating and ischemia is unlikely.						0.698	
31	After deflating the tourniquet cuff, I check the site of incision (e, g, bandaging and drain) for the extent of bleeding.						0.419	
32	After removing the tourniquet cuff, I examine the patient’s skin (e.g. temperature, color, and injuries) where the cuff was placed.						0.489	
**Subscale7**: record-keeping and documentation								
33	In the operative report, I write the name of the person who fastened the tourniquet cuff.							0.605
34	In the operative report, I write the model, registration code, type of cuff, and periodic calibration of the tourniquet.							0.491
35	In the operative report, I write the site of the cuff and the type of padding used under the cuff.							0.594
36	In the operative report, I record the limb occlusion pressure or systolic blood pressure.							0.644
37	In the operative report, I record the tourniquet pressure used during surgery.							0.719
38	In the operative report, I record the patient’s skin condition before and after application of the tourniquet cuff.							0.598
39	In the operative report, I record the times of inflation and deflation of the cuff.							0.598
40	In the operative report, I record the status of blood circulation in the patient’s limb before and after application of the tourniquet cuff by checking the patient’s pulse, capillary refill time, and color and temperature of the limb.							0.501
41	If I notice ischemia in the limb after application of the tourniquet cuff, I record it in the operative report.							0.582

Evaluation of the internal consistency of the scale resulted in a Cronbach’s alpha of 0.85. Using the split-half method, the researchers found the correlation between the even-numbered and odd-numbered items to be a satisfactory 0.72. After applying the test-retest method, the researchers found the intraclass correlation between the two tests to be 0.95, which verified the stability of the scale.

### Responsiveness

No participant had the highest or lowest possible score, proving no floor or ceiling effects.

### Scoring

In the present study, all the items on the instrument were scored positively. Using a 5-point Likert scale for the 41items, the minimum total score is 41 (41 × 1 = 41) and the maximum is 205 (41 × 5 = 205). The cut-off point was determined using the subtraction of the maximum score from the minimum divided by three and it was found to be about 55. This amount was added to the minimum score (41) to determine the divisions. Thus, the score range in the lower third (indicating the least compliance with safety standards) equaled 41 to 95, the middle third (moderate compliance with safety standards) equaled 96 to 150, and the upper third (greatest compliance with safety standards) was from 151 to 205.

## Discussion

The present study was an attempt at developing and validation of a comprehensive and standard instrument for measuring safety and work standards in applying pneumatic tourniquets for all the members of the surgical team in clinical environments. In the present scale, five items in the first subscale address the protocols for testing the functioning of pneumatic tourniquets. These protocols are intended to increase the lifetime and efficacy of these medical devices [33]. Three items (items 17, 32, and 38) are related to considerations on examining patients’ skin to avoid burns. Studies report that chemical burns are a preventable complication in application of pneumatic tourniquets [34–36].

Five items (items 10, 11, 12, 33, and 35) are about the significance of selecting the right cuff and padding under the tourniquet cuff. Injury to the nerves is more likely at the edges of the cuffs than in the middle of the cuff. Also, the upper limbs, where the nerves are closer to the surface of skin tissues, are more prone to injury than the lower limbs [6].

Ten items (items 23, 24, 25, 26, 27, 28, 29, 36, 37, and 39) deal with the pressure and time of application of pneumatic tourniquets, indicating the significance of these two factors in the present scale. According to a systematic review, nerve injuries associated with tourniquets can occur at a wide range of times and pressures. Therefore, surgeons must consider them as a potential complication [11]. There is a direct correlation between feeling pain and the duration of using tourniquets, and the complication is more likely to occur when patients experience local numbness [7]. Several studies have suggested using limb occlusion pressure (LOP) to calculate the pressure of tourniquets. LOP is the amount of pressure exerted by the tourniquet to stop the flow of distal blood as determined by a Doppler probe. In general, the amount of this pressure is higher than systolic blood pressure (SBP). According to the guidelines issued by AORN, during surgery, tourniquets must be inflated to a pressure more than LOP, allowing for a safety margin in case of fluctuations in blood pressure. If LOP is less than 130 mmHg, the safety margin is 40 mmHg. If LOP is between 131 and 190 mmHg, the safety margin is 60 mmHg, and if LOP is more than 190 mmHg, the safety margin is 80 mmHg. For children, a safety margin of 50 mmHg has been recommended [37, 38].

In the instrument developed by Yalcinkaya et al. [19], the items were extracted from a review of five sources, while the pool of items in the present study was the result of an extensive literature review. Moreover, unlike the present study, this instrument does not include items on checking the working condition of the tourniquet, cases where tourniquets cannot be used, considerations about selecting the right cuff, checking the patient’s pulse in the limb before and after applying the tourniquet, checking for leakage of the prep solution under the cuff, post-tourniquet application considerations, and the principles of documenting observations.

The instrument developed by Boya et al. [16] fails to address such factors as checking the working condition of the device, selecting the right cuff and manner of deflating it, measures intended to avoid burns to patients, informing the surgical team of the length of using the tourniquet, measures to be taken after application of the tourniquet, and the principles of recording observations, all of which were dealt with in the present study. In addition, the items of this instrument were developed based on the views of the five authors of the article and the scale was not subject to validity and reliability tests. One of the strengths of this instrument is that it addresses the time of using preventive antibiotics before application of tourniquets, maximum duration of using tourniquets for children, causes of bleeding despite application of tourniquets during surgery, and measures to be taken when the duration of inflation of a tourniquet increases. All of these considerations are dealt with in the present scale too.

Unlike the present study, the instrument developed by Albaker et al. [10] does not address many aspects of application of pneumatic tourniquets, including checking the condition and location of the device, infection control in case of the cuff’s contamination by blood and other fluids, cases where tourniquets cannot be used, selecting the right cuff, informing the surgical team about the pressure of the tourniquet, the angle of the upper and lower limbs at the time of exsanguination, checking the patient’s vital signs before and after deflation of the cuff, examining the patient’s skin and distal pulses, and the principles of documenting observations. Moreover, the validity of this instrument was not measured and only 20 subjects were involved in validation of its reliability.

The instrument developed by Ajibade et al. [11] not only fails to address the various aspects of applying pneumatic tourniquets, but did not go through psychometric evaluation. In comparison with the scale developed by Cunningham et al. [20], the present instrument not only covers all the items on the former scale, but contains 10 questions on the pressure and duration of using pneumatic tourniquets examined from different aspects.

All the scales developed in previous studies were designed to measure orthopedic surgeons’ knowledge of pneumatic tourniquets. The instrument developed in the present study can be used to assess the performance of all the members of the surgical team in applying pneumatic tourniquets, which increases the utility and scope of the scale. Moreover, unlike the previous studies in which the validity of the scales was not measured, careful evaluation of the validity and reliability of the present scale resulted in a robust instrument.

Before a new instrument for measuring a concept can be developed, the various aspects of the concept in question need to be carefully explored through extensive review of literature and counsel of experts. Overall, the above-mentioned instruments have been developed based on a review of a few articles, one guideline, or the views of the authors; thus, they fail to address all the aspects of safety and work standards in using pneumatic tourniquets. In the present study, however, the researchers conducted an extensive review of literature to create the pool of items and consulted a panel of experts consisting of many members. Moreover, the previous scales did not go through the stages of instrument development and psychometric evaluation. In the present study, in addition to qualitative and quantitative face and content validity and construct validity, the researchers measured the reliability and consistency of the instrument. In all the stages of psychometric evaluation of the scale, i.e. face validity (measured qualitatively and quantitatively with 15 individuals), content validity (measured qualitatively and quantitatively with 15 individuals), construct validity (300 individuals), and reliability (30 individuals), sampling adequacy was observed.

The present study is an innovation in Iran and in the world in the field of developing and testing the psychometric properties of an instrument for measuring safety standards in application of pneumatic tourniquet. The impact scores of all the 41 items were above 1.5, confirming the high significance of the items. With regard to validity, the CVRs and CVIs of all the items were greater than 0.49 and 0.79 respectively. The S-CVI/UA and S-CVI/Ave of the scale were found to be 0.95 and 0.99 respectively, which verifies that the scale possesses very good content validity. In this study, most of the items had factor loading values of more than 0.5. There is no clear consensus on what constitutes an acceptable factor loading value. Some researchers suggest that factor loadings of 0.3 or higher are acceptable. Others suggest that factor loadings of 0.4 or higher are acceptable. However, in general, factor loadings of 0.2 or lower are considered poor. For example, Stevens (2012) suggested that the value of a factor loading should be greater than 0.4 for interpretation purposes [39], whereas Hair et al. (2016) argued that all standardized factor loadings should be at least 0.5 and, ideally, at least 0.7 [40]. In this study the factor loading of some of the items were between 03 and 05 and none of the items was smaller than 0.3. Because these items were essential to measuring pneumatic tourniquet safety and work standards as judged by the panel of experts, they were retained. In the present study, construct validity was determined using EFA. Three main questions arise when conducting an EFA: (1) The method of factor extraction; (2) How many factors to settle on for a confirmatory step; and (3) Which factor rotation should be employed. On the other hand, there is no clear distinction between EFA and CFA in most attempts at factor analysis and the two fall on a continuum running from exploration to confirmation [41]. EFA fulfilled the purpose of the study, but it is suggested that further evaluation and validation of the scale’s properties be carried out to cross-validate and extend its utility and psychometric evidence.

Using the split-half method, the researchers found the correlation between the items to be 0.72, which indicates that the internal consistency of the scale is satisfactory. Applying the test-retest method resulted in an interclass correlation coefficient of 0.95, confirming the stability of the scale.

To develop the present scale, the researchers consulted a panel of experts familiar with application of tourniquets in Iran. Therefore, the findings of the study may not be transferrable to all countries. However, as the items were generated after a comprehensive review of literature, the present scale seems to have the potential to be used in other societies as well. It is suggested that future research validate this instrument in other settings. By employing the present scale in other contexts, other researchers can study the patient outcomes of using this scale regarding the consequences of pneumatic tourniquets.

## Conclusion

The developed scale in the present study is adequately valid and reliable and can be used in operating rooms to measure surgical teams’ knowledge and practice with regard to pneumatic tourniquet safety standards. Evaluation of the operating room personnel’s performance with regard to applying these devices can help reduce the hazards associated with using pneumatic tourniquets and improve the surgical process. By benefiting from valid and up-to-date scientific sources, the present scale addresses different aspects of the application of pneumatic tourniquets and enables all the members of the surgical team to provide better quality medical services. In addition, this scale can be employed by other researchers and managers of medical centers to identify the existing risk factors and subsequently develop educational programs or introduce new policies to reduce or eliminate them.

## Data Availability

No datasets were generated or analysed during the current study.
